# Grafting Going Green: Toward a Sustainable Preparation of Organic–Inorganic Hybrid Materials

**DOI:** 10.1021/acs.jpcb.2c04243

**Published:** 2022-09-07

**Authors:** Julio Cesar Fernandes P. Brito, Fabio Travagin, Ivana Miletto, Giovanni Battista Giovenzana, Enrica Gianotti

**Affiliations:** † Dipartimento per lo Sviluppo Sostenibile e la Transizione Ecologica (DISSTE), Università del Piemonte Orientale, Piazza Sant’Eusebio 5, I-13100 Vercelli, Italy; ‡ Dipartimento di Scienze del Farmaco (DSF), Università del Piemonte Orientale, Largo Donegani 2, I-28100 Novara, Italy

## Abstract

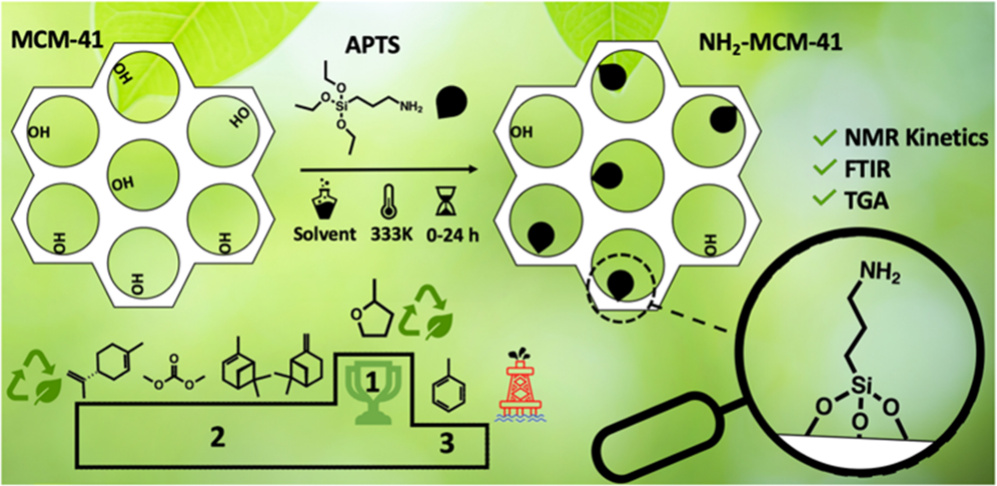

Organic–inorganic hybrid materials find many applications in catalysis, nanotechnology, electronics, and many others. Grafting organic functionalities on inorganic supports is one of the most used methods for their preparation. Toluene is the solvent of choice for the grafting reaction, but it is fossil fuel-derived and not devoid of toxic effects. In this work, we explore the use of sustainable alternatives, *i.e.*, (+)-α-pinene, (−)-β-pinene, dimethyl carbonate (DMC), (+)-limonene, and 2-methyl-tetrahydrofuran (MeTHF), as solvents for grafting. The grafting reaction between 3-aminopropyltriethoxysilane (APTS) and mesoporous ordered silica (MCM-41) was selected as a model for this study. A comparison of the rate of the grafting reaction in different solvents is reported. The resulting hybrid materials were analyzed by Fourier-transform infrared (FTIR) spectroscopy and thermogravimetric analysis (TGA) and compared to the reference material prepared in toluene. MeTHF proved to be the best sustainable alternative to toluene for model grafting, providing a comparable product in a significantly shorter reaction time.

## Introduction

The double soul (organic–inorganic) of hybrid materials is spreading throughout a constantly increasing number of fields of chemistry.^1^ Hybrid materials are gaining growing importance due to the wide tunability of their structure and properties: changes in the organic or in the inorganic components or in the interface between them will deeply influence the features of the resulting material.^2,3^ Hybrid materials find widespread applications in medicine, electronics, photovoltaic technology, tire construction, optics, catalysis, and many others.^4^ In particular, in heterogeneous catalysis, hybrid materials are able to catalyze the most diverse reactions,^5^ ranging from acid–base-catalyzed transformations to cross-couplings and multicomponent reactions and even multiple catalytic sites for multistep transformations.^6^ Homogeneous catalysts generally show higher specific activities, but heterogeneous catalysts can be easily removed from the reaction mixture and can be recycled.^7^ Since many catalysts, such as transition-metal complexes or organocatalysts for asymmetric synthesis, are usually expensive or toxic, their complete recovery from the reaction mixture and their recycling is particularly important for the sustainability of the process and for the safety of the product.^8^

**Figure 1:**
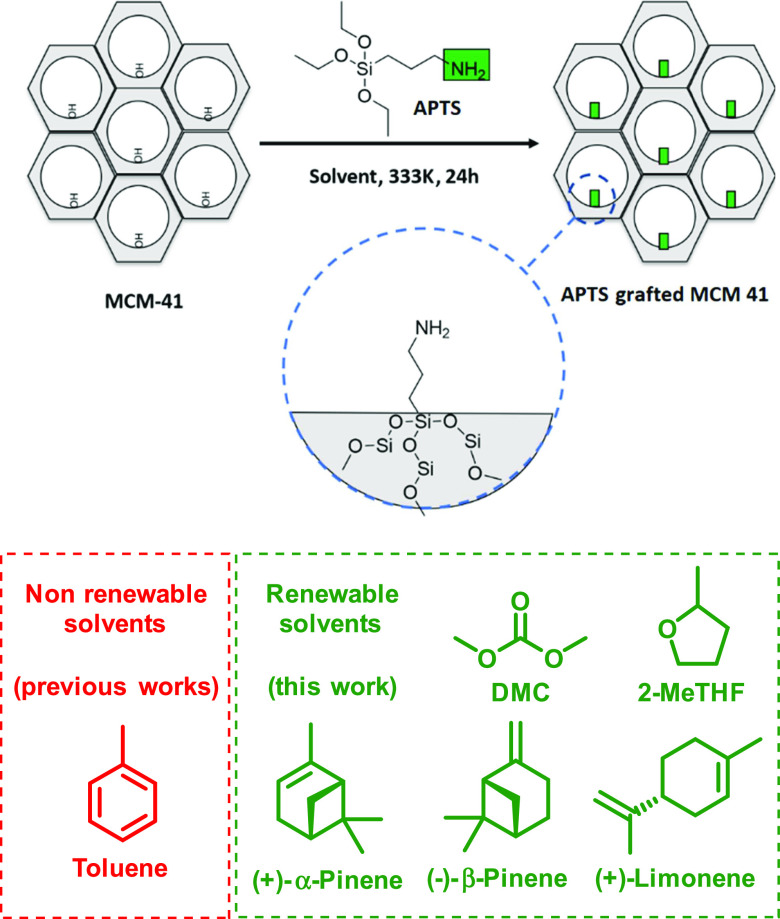
Grafting APTS on MCM-41.

Among the various hybrid catalysts, mesoporous inorganic ordered silica (MCM-41) functionalized with pendant amino groups has been widely studied and used as a basic catalyst in many reactions, such as hydroformylation,^9^ Knoevenagel condensation, heterocyclic chemistry,^10^ cross-coupling reactions, and many others.^11^ 3-Aminopropyltriethoxysilane (APTS) is usually grafted on the silanol groups present in the inner walls of MCM-41 to synthesize these hybrid materials (Figure 1). The grafting of organic moieties to a solid surface is a postsynthetic versatile tool for surface modification and functionalization. Following this functionalization method, it is possible to introduce one or more organic functions obtaining single- or multisite hybrid catalysts.

The grafting reaction is normally run using a solvent to solubilize the organic reactant(s), disperse the support particles, reduce the viscosity of the reaction, and moderate eventual exothermic reactions.

The choice of the solvent is crucial as it must be inert and respectful of the structural integrity of the inorganic support and of the organic molecules, the latter usually being highly reactive, such as APTS.^12^ Moreover, the solvent represents the larger portion of the reaction mass, with a major impact on the safety and the environmental impact of the process.

For these reasons, toluene is generally employed in grafting procedures.^13,14^ Toluene has an acceptable boiling point, allowing the reaction mixture to be heated up to 111 °C if necessary but can be easily removed from the material, afterward. Moreover, toluene is cheap and widely available on the market. However, toluene is a nonrenewable solvent, deriving from the catalytic reforming of light petroleum fractions as well as posing a significant danger to human health.^15,16^ The search for greener solvents to be used in the grafting method represents nowadays a challenge toward more sustainable synthetic methodologies. In this article, we investigate the use of green and sustainable solvents alternative to toluene for the grafting reaction, studying their effect on the resulting hybrid materials.

## Materials and Methods

APTS was purchased from TCI Europe and used without further purification. MCM-41 was purchased from Sigma-Aldrich (Milano, Italy). All solvents were purchased from Merck or TCI Europe and dried overnight before use over 4 Å molecular sieves.

### Grafting Reaction

MCM-41 (50.7 ± 2.1 mg) was weighted inside 10 mL polypropylene test tubes, and the solvent (3 mL) and APTS (30 μL) were added. The test tubes were stoppered and heated at 60 °C with stirring. The grafting reaction was followed by ^1^H NMR to study the kinetics and the APTS grafting efficiency. For each sampling, 50 μL of the supernatant was taken and diluted in 500 μL of pyrazine solution (0.05 M in CDCl_3_). Pyrazine was used as the internal standard for the NMR analyses, integrating its singlet at 8.58 ppm. The concentration of APTS was measured by the integral of the triplet peak of the methylene in α to the amino group of APTS at 2.72 ppm, according to eq S1 (Supporting Information).^17^ At the end of the grafting reaction, the mixture was then cooled to room temperature, filtered under vacuum, and the solid hybrids were washed with the reaction solvent. The hybrids were then dried in an oven at 80 °C for 5 h to remove the residual solvent.

### Characterization

^1^H NMR spectra were recorded at 400 MHz on a Bruker Avance Neo 400 spectrometer operating at 25 °C (32 scans, 5 s delay). Chemical shifts are reported in ppm with the protic impurities of the deuterated solvent as the internal reference. FTIR spectra of self-supporting pellets were collected after thermal treatment at 180 °C for 1 h under vacuum conditions (residual pressure <10^–4^ mbar) using a Bruker Equinox spectrometer equipped with a pyroelectric detector (deuterated triglycine sulfate (DTGS) type) with a resolution of 4 cm^–1^. FTIR spectra were normalized with respect to the pellet density. Thermogravimetric analysis (TGA/DTGA) of the samples was performed under a N_2_ flow (20 mL/min) with a Setaram LABSYS evo instrument, heating from 30 to 800 °C at 5 °C/min.

## Results and Discussion

Our investigation was focused on solvents deriving from biomasses and/or renewable sources, with an inertness comparable to toluene. The solvents used in this work are shown in Figure 1; their properties and the acronyms of the hybrid organic–inorganic materials obtained are listed in Table 1.

**Table 1:** Solvents Used in the Grafting Reaction and the Corresponding Hybrid Materials

solvent	boiling point (°C)	dielectric constant	flash point (°C)	hybrid material #
toluene	111	2.38^23^	4	Hyb_1
MeTHF^a^	80	6.97^23^	–10	Hyb_2
(+)-limonene	176	2.37	45	Hyb_3
DMC^b^	90	3.09^23^	17	Hyb_4
(−)-β-pinene	165	2.50	36	Hyb_5
(+)-α-pinene	157	2.18	33	Hyb_6

a (±)–2-Methyltetrahydrofuran.

b Dimethyl carbonate.

To the best of our knowledge, few reports on the effect of the solvent on the grafting reaction and on the resulting hybrid material have been reported^18^ and even no systematic study on the use of green or sustainable solvents. An exception must be made for the use of alcohols, usually methanol and ethanol, as solvents for the grafting reaction. Their use is safe for the operator, and they are generally considered green solvents, but they are not inert toward both the support and the organic reactants, and for these reasons, alcohols were not considered in this work.^19,20^

In this work, we focus our attention on solvents derived from biomasses or prepared by green synthetic procedures on a large scale. (±)-2-Methyltetrahydrofuran (MeTHF) derives from the catalytic hydrogenation of furfural, obtained from food industry wastes.^21^ The natural acyclic hydrocarbon (+)-limonene is obtained from the oil derived from citrus fruit peels,^16^ while the isomeric bicyclic (+)-α-pinene and (−)-β-pinene are obtained from pine resin.^22^ The absence of polar functional groups in these monoterpenes ensures an inertness comparable to toluene. Dimethyl carbonate (DMC) is a synthetic solvent prepared by the catalytic oxidative carbonylation reaction between carbon monoxide and oxygen in methanol to yield DMC in high yields.^23^ Their use is consolidated in many industrial processes owing to their safety, low toxicity, and low cost.^16,21−23^

The grafting of APTS on mesoporous MCM-41 is chosen as a model reaction. This choice is based both on the importance of the resulting hybrid materials and on the large literature available mostly using toluene as a solvent.^24,25^ The grafting reaction is followed by ^1^H NMR to study the kinetics and the APTS grafting efficiency. The synthesized hybrids obtained are finally characterized by FTIR spectroscopy and thermogravimetric analysis.

**Figure 2:**
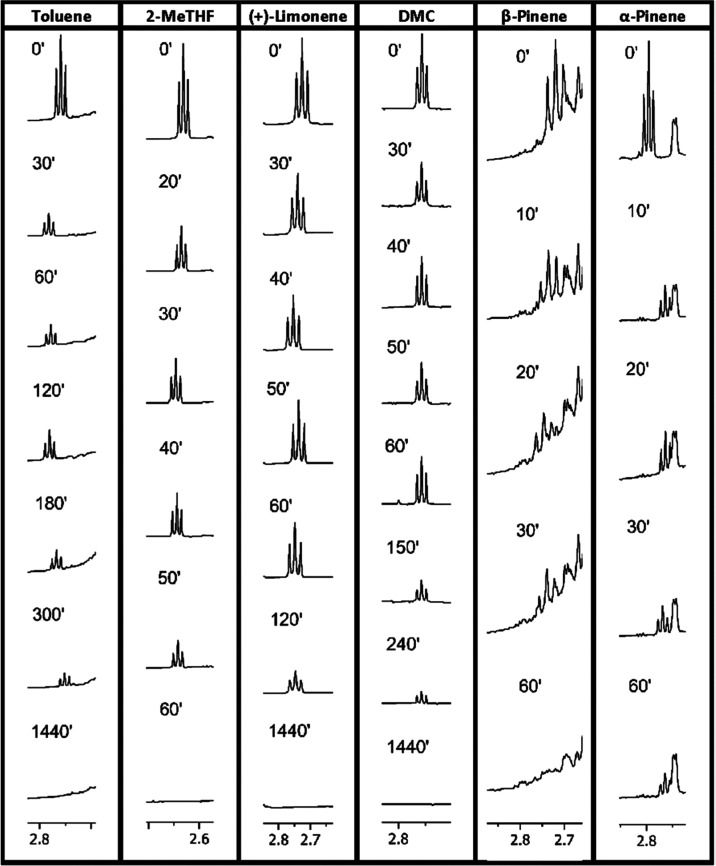
Evolution of ^1^H NMR spectra of APTS as a function of time using different solvents (−CH_2_NH_2_, *T* = 60 °C).

Figure 2 reports the evolution of ^1^H NMR spectra of APTS as a function of time in different solvents; a larger zoom can be observed in Figures S1–S6 in the Supporting Information.

**Figure 3:**
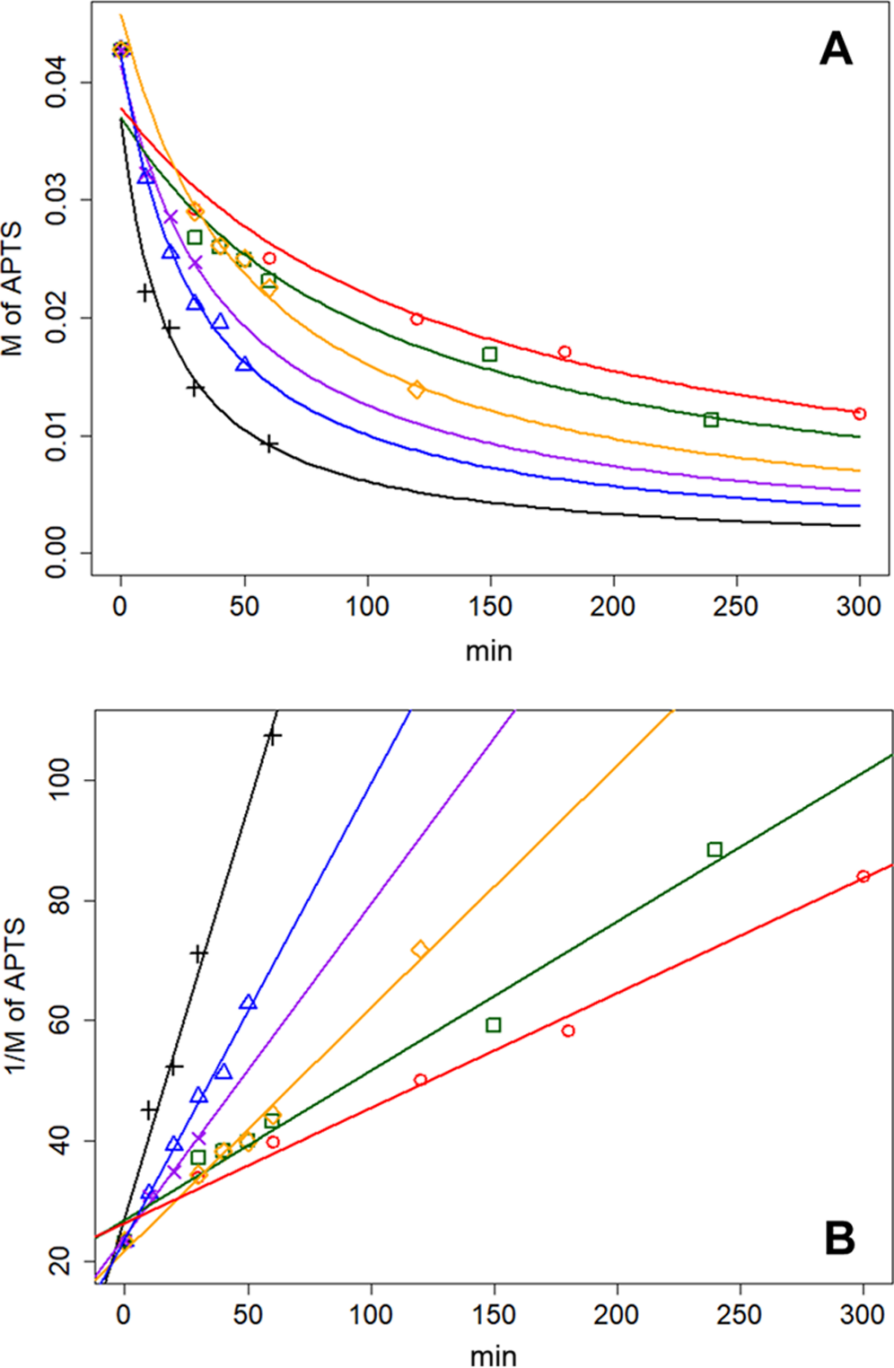
Kinetic profiles of grafting reactions in various solvents fitted in a second-order kinetic and curves thereof (A: nonlinearized, B: linearized): open red circles, toluene; open blue up triangles, 2-methyltetrahydrofuran, open orange diamonds, (+)-limonene; green squares, dimethyl carbonate; purple crosses, (−)-β-pinene; and black plus symbols, (+)-α-pinene.

The residual concentration values of APTS were used to determine the kinetic profile of the grafting reaction in the different solvents cited above (eq S1).^26^ The kinetic profiles of the grafting reactions in different solvents, fitted in a second-order kinetic,^26−28^ are reported in Figure 3. For the sake of clarity, the complete set of data is available, along with the corresponding slope and *R*^2^ values, in Figures S7–S12 (Supporting Information). It is worth noting that the first-order kinetic provided a good fitting too for these data, but *R*^2^ values were always lower than those obtained with the second-order kinetic.

**Figure 4:**
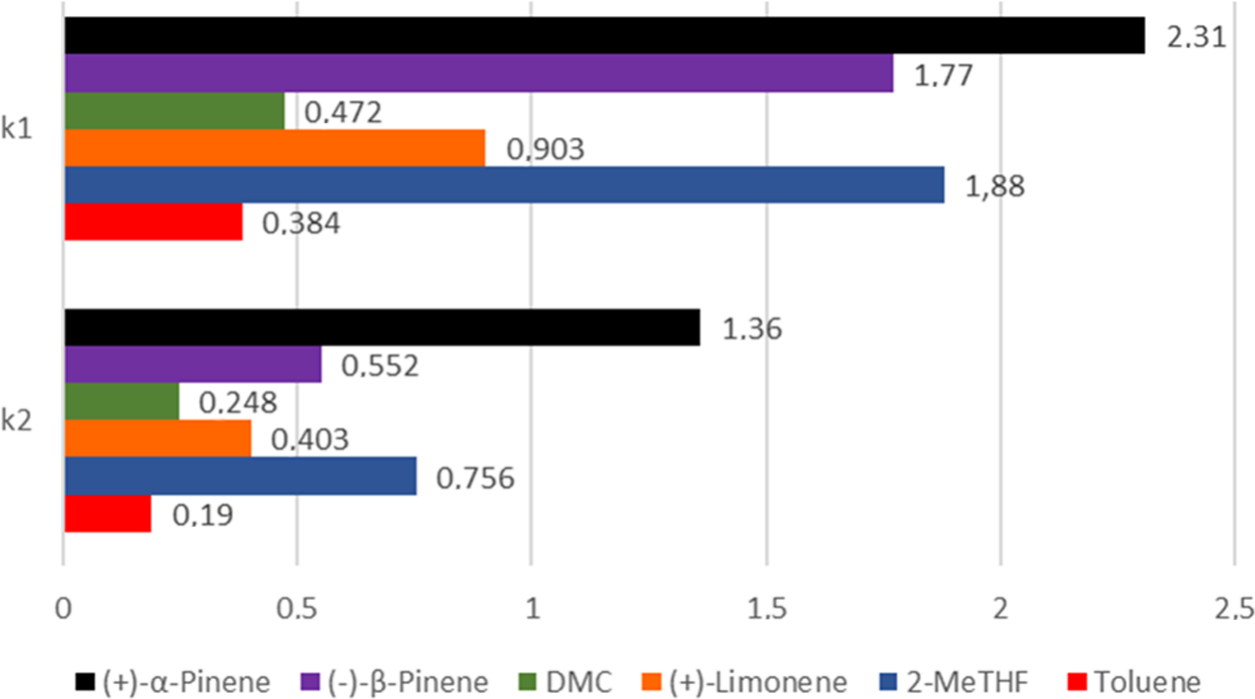
Grafting kinetic parameters in various solvents, *k*_1_ = first-order kinetic constant [×10^–2^ min^–1^], *k*_2_ = second-order kinetic constant [*M*_APTS_^–1^ min^–1^].

The kinetic parameters of the grafting reactions are summarized in Figure 4. As shown in Figures 2–4, the fastest kinetics for the grafting reaction between APTS and MCM-41 was exhibited by (−)-α-pinene, followed by MeTHF, (−)-β-pinene, (+)-limonene, dimethyl carbonate, and toluene.

Kinetic monitoring provides preliminary information on the reaction progress in the selected solvents. However, the kinetic data are not sufficient for a complete description of the grafting methodology, as the consumption of APTS could be due to unwanted reactions with the solvent or with adventitious humidity.

**Figure 5:**
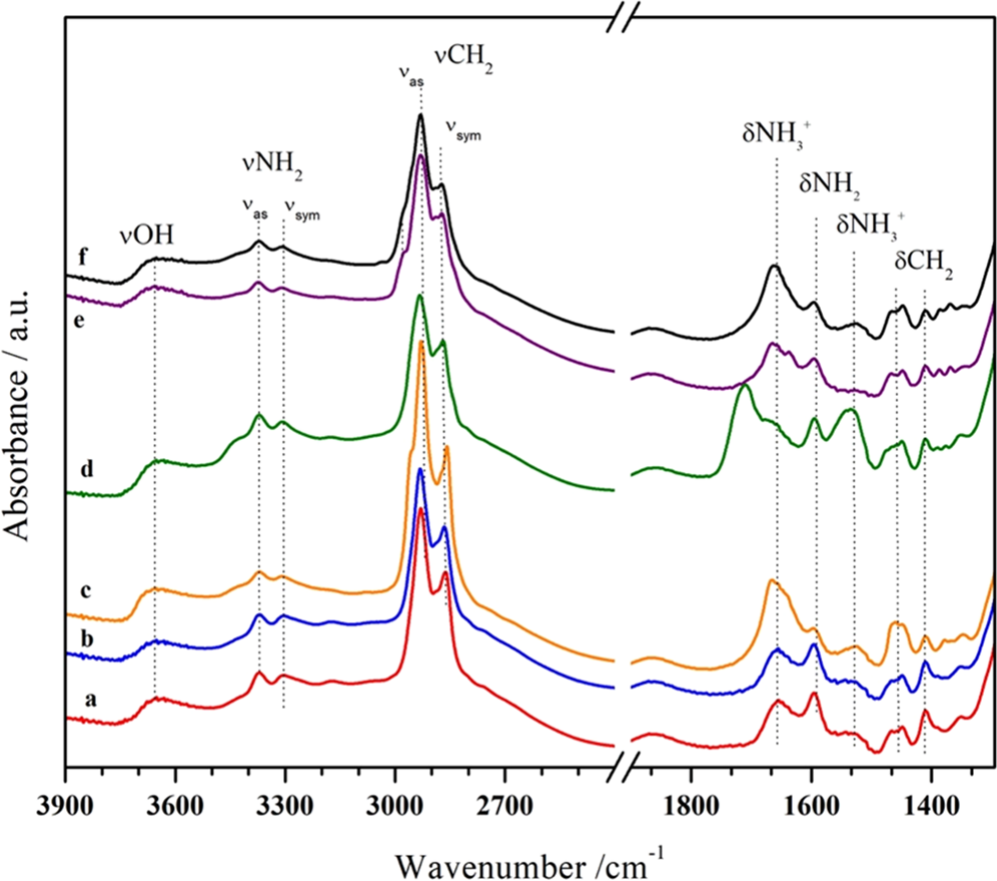
FTIR spectra of the solid hybrids obtained in different solvents upon thermal treatment for 1 h at 180 °C to remove physisorbed water: Hyb_1 (curve a, red), Hyb_2 (curve b, blue), Hyb_3 (curve c, orange), Hyb_4 (curve d, green), Hyb_5 (curve e, purple), and Hyb_6 (curve f, black).

Therefore, FTIR spectroscopy and TGA are used to highlight that APTS is efficiently grafted on MCM-41 (Figure 5).

In the spectra of all hybrids, the typical signal of the O–H stretching mode of free silanols at 3745 cm^–1^ (Figure S13 in the Supporting Information) is almost disappeared, confirming the success of the APTS grafting, and a broad feature at lower wavenumbers is visible due to the OH interaction *via* H-bond.^3^

Furthermore, the characteristic signals of the asymmetrical and symmetrical stretching modes of the amino group (−NH_2_, 3373, 3305 cm^–1^) and of the methylene groups (−CH_2_, 2943 and 2870 cm^–1^) belonging to APTS are observed.^29^ At lower wavenumbers, signals at 1653 and *ca.* 1550 cm^–1^ are due to the bending mode of NH_3_^+^ species, suggesting an acid–base equilibrium between some amino groups and surface SiOH, while the signal at 1590 cm^–1^ is due to the asymmetric bending mode of free −NH_2_ groups.^30,31^

The CH_2_ bending modes are also visible at 1449 and 1400 cm^–1^. In the case of Hyb_3 to Hyb_6 spectra, additional signals below 1800 cm^–1^ are ascribed to residual solvent molecules adsorbed on the solid surface. Among all, the FTIR spectra of Hyb_1 and Hyb_2 obtained with toluene and MeTHF are almost superimposable, suggesting an equivalent grafting in these two solvents with no significant interaction with either the solid silica or the grafted APTS. In fact, the signals of free NH_2_ are the major contributors to the FTIR spectra at both high and low wavenumbers and only a fraction of amino groups can be protonated by silanols. However, even if they are formed, these protonated species are not stable in the presence of water or other solvents; this means that the interaction between silanols and amino groups is weak and free amino groups can be restored easily.^31^

**Figure 6:**
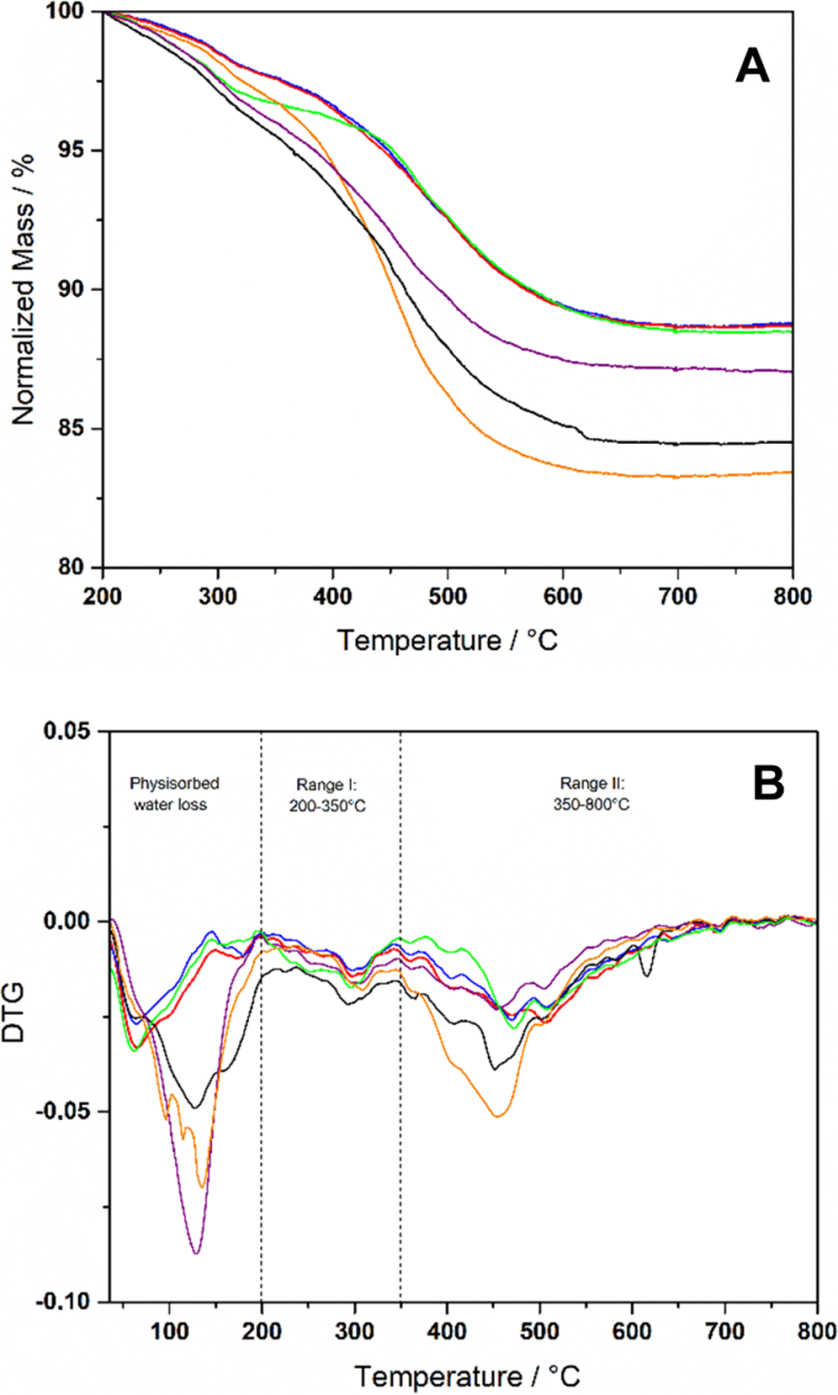
TG (A) and DTG (B) curves of MCM-41 with the APTS anchored in different solvents: Hyb_1 (curve red), Hyb_2 (curve blue), Hyb_3 (curve orange), Hyb_4 (curve green), Hyb_5 (curve purple), and Hyb_6 (curve black).

The thermogravimetric (TG) profiles of the different hybrids are reported in Figure 6A; TG data are normalized to the weight of the dry sample, *e.g*.: after the weight loss in the range of 30–200 °C, due to the removal of physisorbed water. The analysis of the derivative (DTG) curves (Figure 6B) helps in identifying two main regions in the decomposition profile of all of the hybrids.

The weight loss in the first region, from 200 to 350 °C, is generally ascribed to the condensation of silanol groups and consequent water loss.^30,32^ In the DTG curve of Hyb_1 and Hyb_2, a not well-resolved two-component endothermic peak is present in the 350–800 °C range. The weight loss in this region is associated with the decomposition of bound phases and is used for the estimation of the amount of grafted aminopropyl groups, reported in Table 2. In particular, the APTS content of Hyb1 and Hyb2 is very close, highlighting the success of using MeTHF as an alternative solvent to toluene for the grafting method.

**Table 2:** Weight Loss (Δwt %) Calculated from the TG Analysis

	Δwt (%)	
sample	range *I*^a^	range II^b^
	200–350 °C	350–800 °C
Hyb_1	2.44	8.86
Hyb_2	2.37	8.87
Hyb_3	3.23^c^	13.33^c^
Hyb_4	3.30^c^	8.23^c^
Hyb_5	3.96^c^	8.99^c^
Hyb_6	4.49^c^	11.01^c^

a Weight loss due to dehydroxylation between 200 and 350 °C.

b Weight loss due to the elimination of APTS-derived groups between 350 and 800 °C.

c Quantification affected by the residual presence of a solvent.

In all of the other hybrid materials, DTG curves show a much complex and broader endothermic peak, in the 350–800 °C range. In these instances, the weight loss can be ascribed to the decomposition of both aminopropyl chains and residual solvent molecules, whose presence is already evidenced by FTIR. For this reason, the quantification of the amount of grafted aminopropyl groups cannot be accurately estimated in the case of Hyb_3 to Hyb_6.

## Conclusions

In summary, in this work, we investigated potential green and sustainable solvents for the replacement of toluene in the grafting reaction of alkoxysilane moieties to MCM-41, as a model for the preparation of hybrid materials for different applications.

Five cheap, safe, and renewable solvents, *i.e.*, (+)-α-pinene, (−)-β-pinene, dimethyl carbonate (+)-limonene, and MeTHF, have been screened in the APTS grafting on mesoporous silica MCM-41 and compared with the same procedure carried out in toluene as a reference. The kinetic profile in the various solvents was followed by ^1^H NMR and revealed that the reaction in α-pinene and in MeTHF proceeds much faster than in toluene.

FTIR and TGA analyses showed the successful grafting of APTS in all solvents. Nevertheless, when α-pinene, β-pinene, dimethyl carbonate, and (+)-limonene were used, residual solvent molecules were detected even after thermal treatment of the hybrids at 180 °C in vacuum, while no evidence of MeTHF was found in the corresponding hybrid material.

In conclusion, this work demonstrates that MeTHF efficiently supports the model grafting reaction of APTS on mesoporous silica. The corresponding hybrid material is fully comparable to that obtained in classical conditions (toluene, reflux), in milder conditions, and shorter reaction times. As MeTHF comes from renewable sources and is cheap and easily recycled, it emerges among the solvents studied as the most suitable for the preparation of organic–inorganic hybrid materials, a more efficient and sustainable alternative to the currently employed toluene.

## Supporting Information Available

Concentration graphs of grafting reactions (Figures S1–S6); equation used for the calculation of APTS concentration (Equation S1); kinetics of grafting reactions (Figures S7–S12); and FTIR spectrum of MCM-41 (Figure S13) (PDF)
