# Supplementary material for: Grafting Going Green: Toward a Sustainable Preparation of Organic–Inorganic Hybrid Materials
Source: J Phys Chem B. 2022 Sep 7;126(37):7166–71. doi: 10.1021/acs.jpcb.2c04243 (PMC9511484; doi:10.1021/acs.jpcb.2c04243)
Supplement: Supplementary file 1 — jp2c04243_si_001.pdf [file jp2c04243_si_001.pdf]

Supporting Information

for

Grafting Going Green: Towards a Sustainable Preparation of Organic-Inorganic Hybrid Materials

Julio Cesar Fernandes P. Brito,[a] Fabio Travagin,[b] Ivana Miletto,[b] Giovanni Battista Giovenzana[b]* and Enrica Gianotti[a]*

[a] Dipartimento per lo Sviluppo Sostenibile e la Transizione Ecologica (DISSTE), Università del Piemonte Orientale, Piazza Sant'Eusebio 5, I-13100 Vercelli, Italy
[b] Dipartimento di Scienze del Farmaco (DSF), Università del Piemonte Orientale, Largo Donegani 2, I-28100 Novara, Italy

Table of contents

Concentration graphs of grafting reactions 2

Kinetics of grafting reactions 5

FT-IR spectroscopy 7

Concentration graphs of grafting reactions

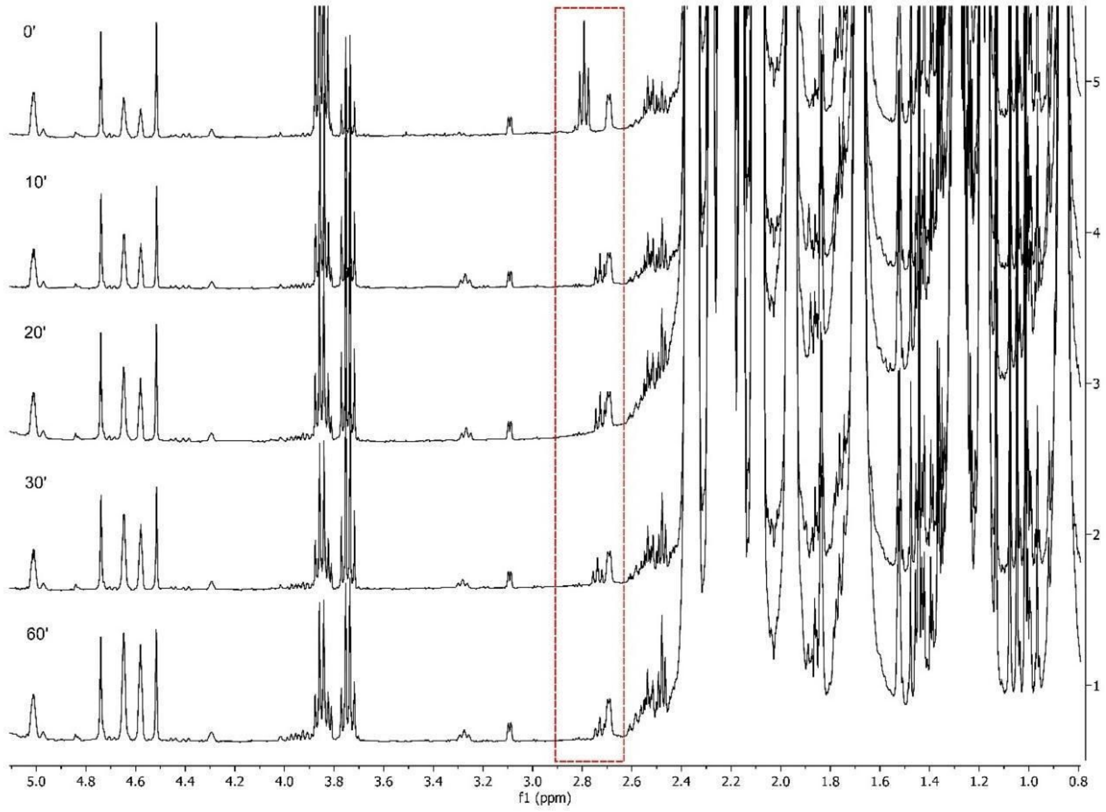

Figure S1. Concentration graph of grafting reaction in α-pinene.

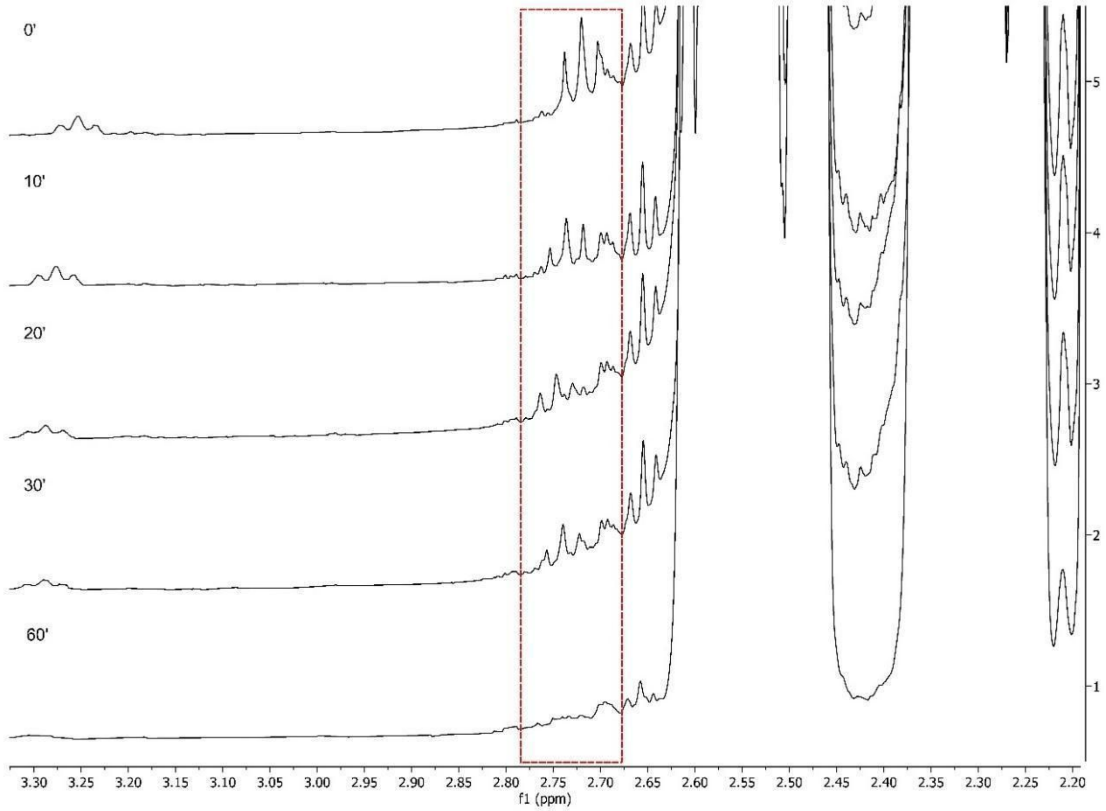

Figure S2. Concentration graph of grafting reaction in β-pinene.

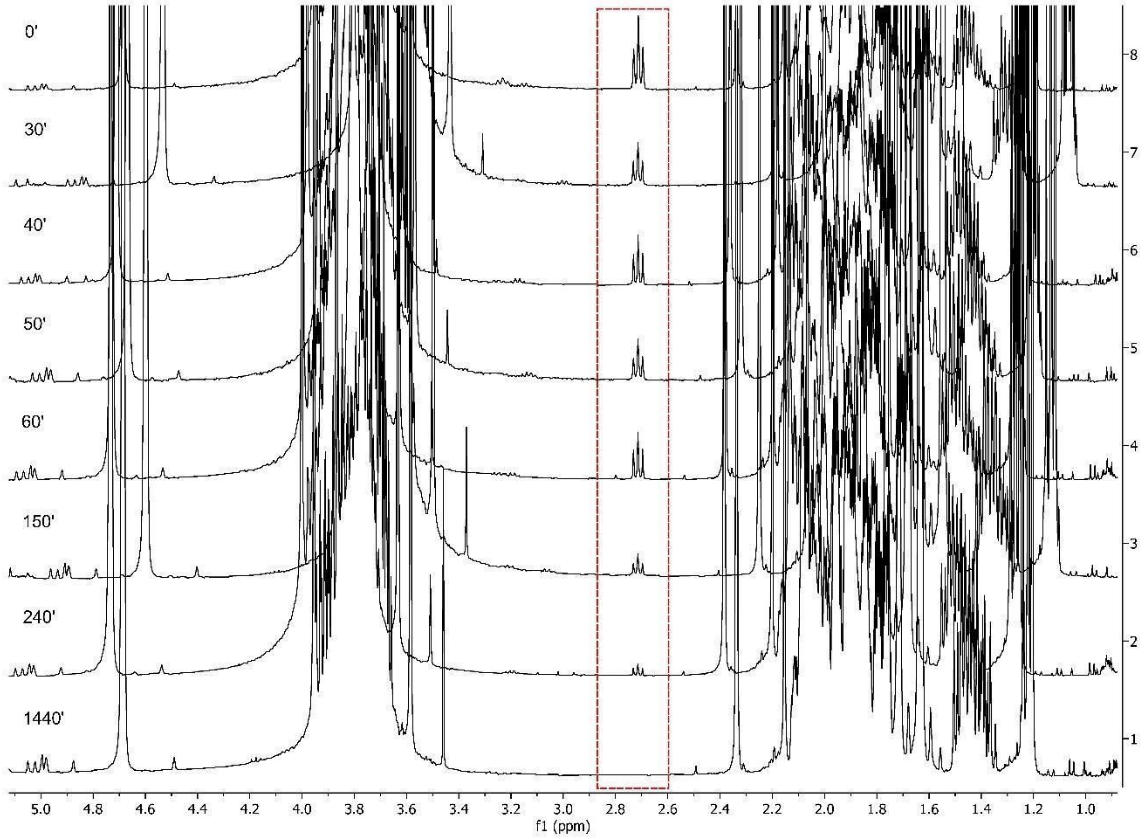

Figure S3. Concentration graph of grafting reaction in dimethyl carbonate.

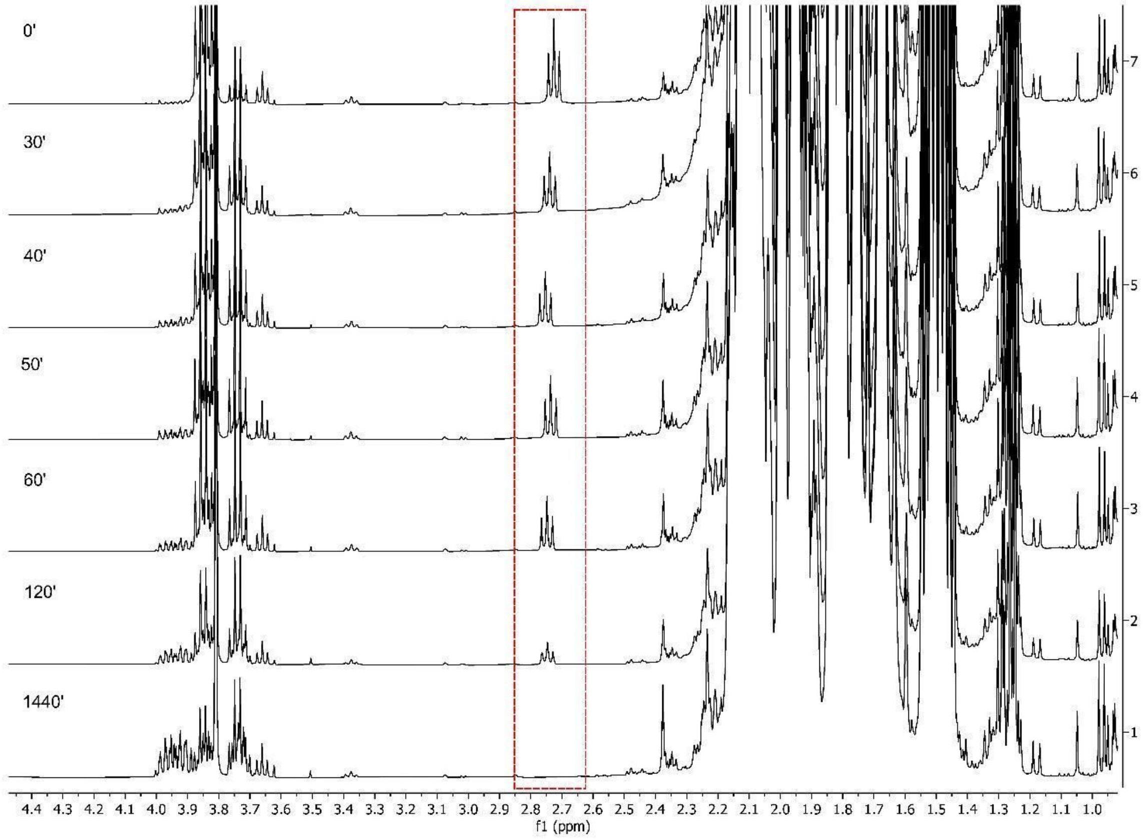

Figure S4. Concentration graph of grafting reaction in (+)-limonene.

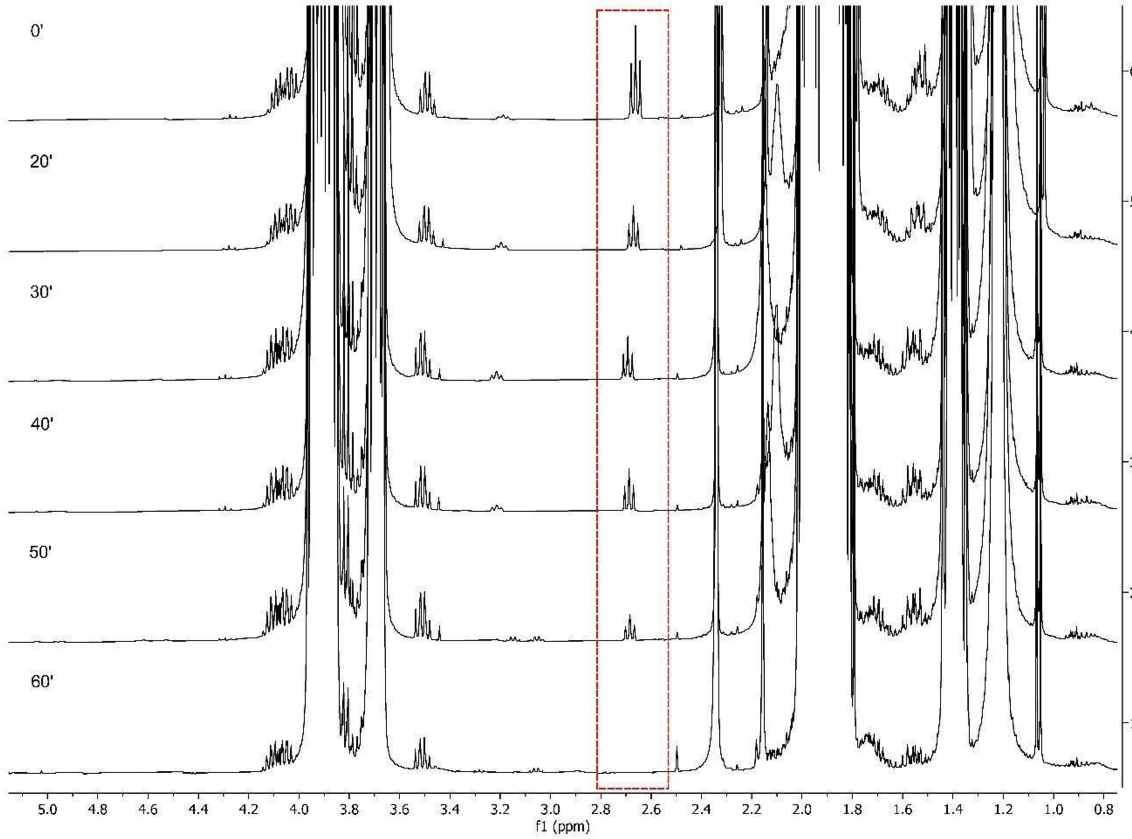

Figure S5. Concentration graph of grafting reaction in 2-methyltetrahydrofuran.

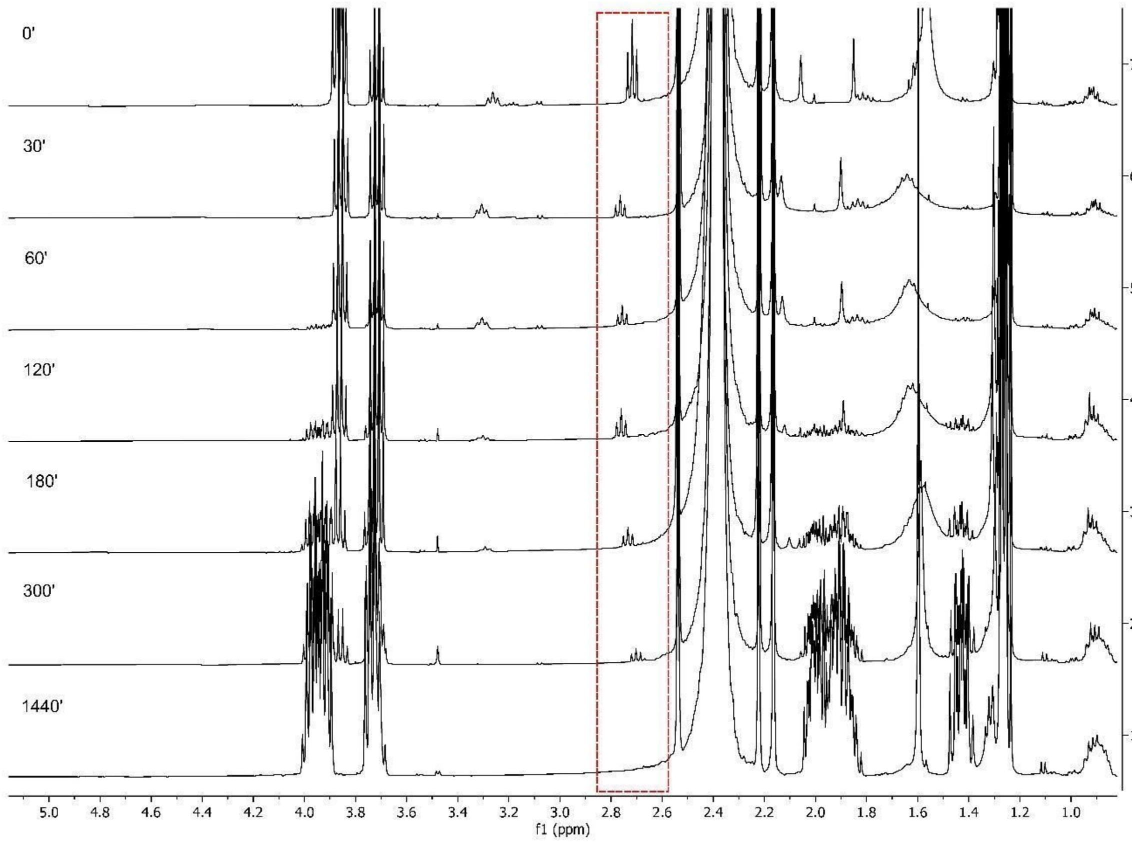

Figure S6. Concentration graph of grafting reaction in toluene.

$$C_{APTES} = \frac{I_{APTS}}{I_{pyrazine}} \cdot \frac{N_{pyrazine}}{N_{APTS}} \cdot 0.05\ M_{pyrazine}$$

Equation S1. *C* = concentration, *I* = integral and *N* = number of nuclei.

Kinetics of grafting reactions

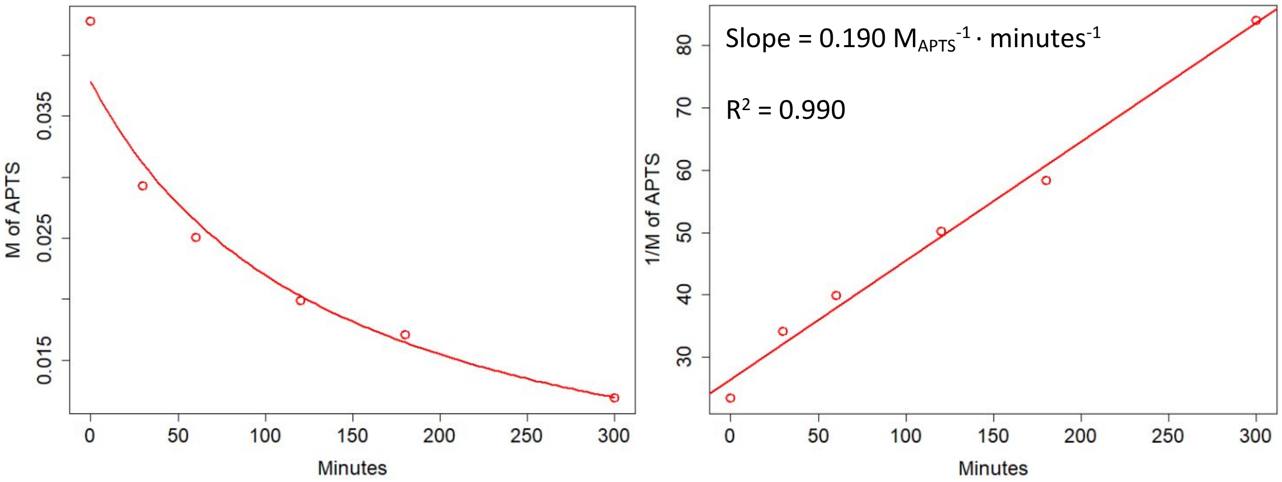

Figure S7. M of APTS consumed over time by grafting reaction in toluene.

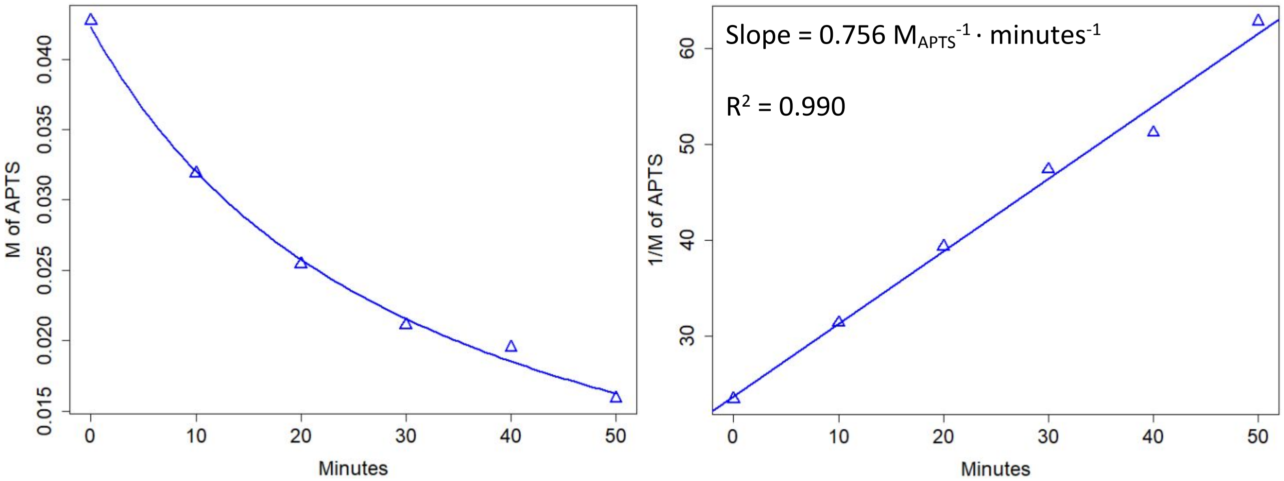

Figure S8. M of APTS consumed over time by grafting reaction in 2-methyltetrahydrofuran.

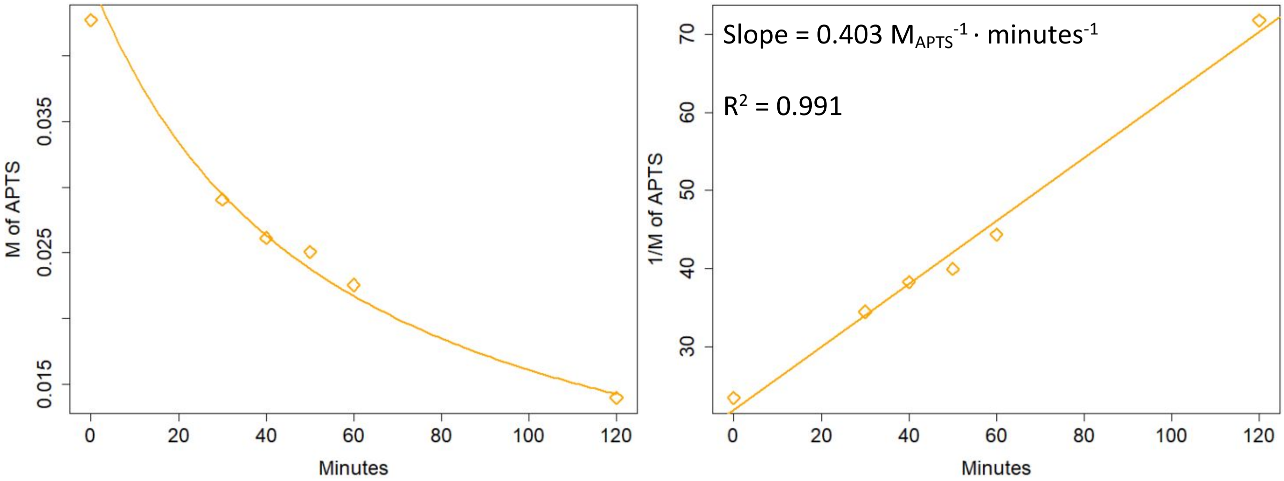

Figure S9. M of APTS consumed over time by grafting reaction in (+)-limonene.

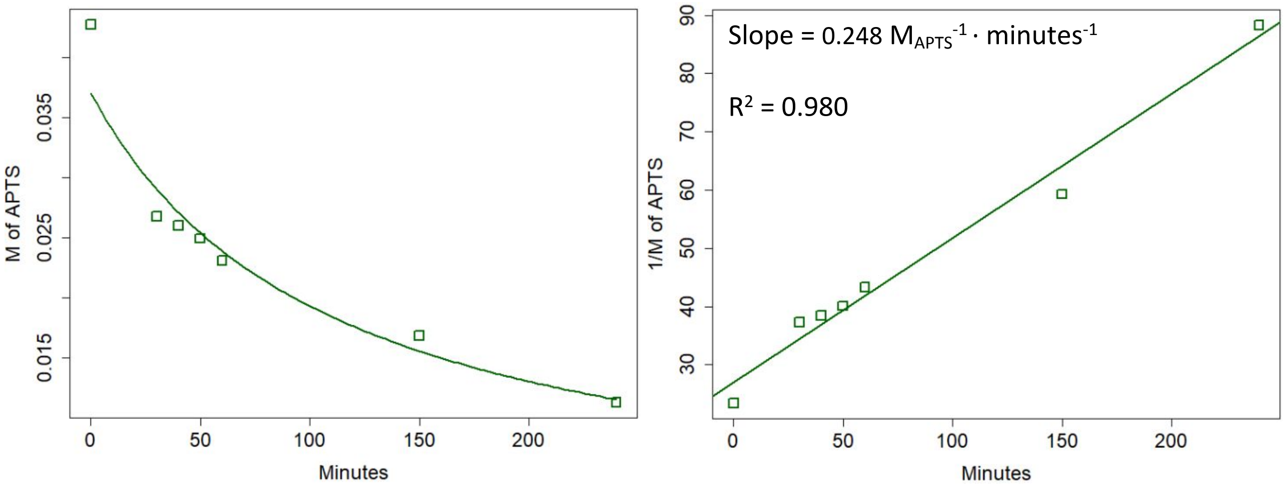

Figure S10. M of APTS consumed over time by grafting reaction in dimethyl carbonate.

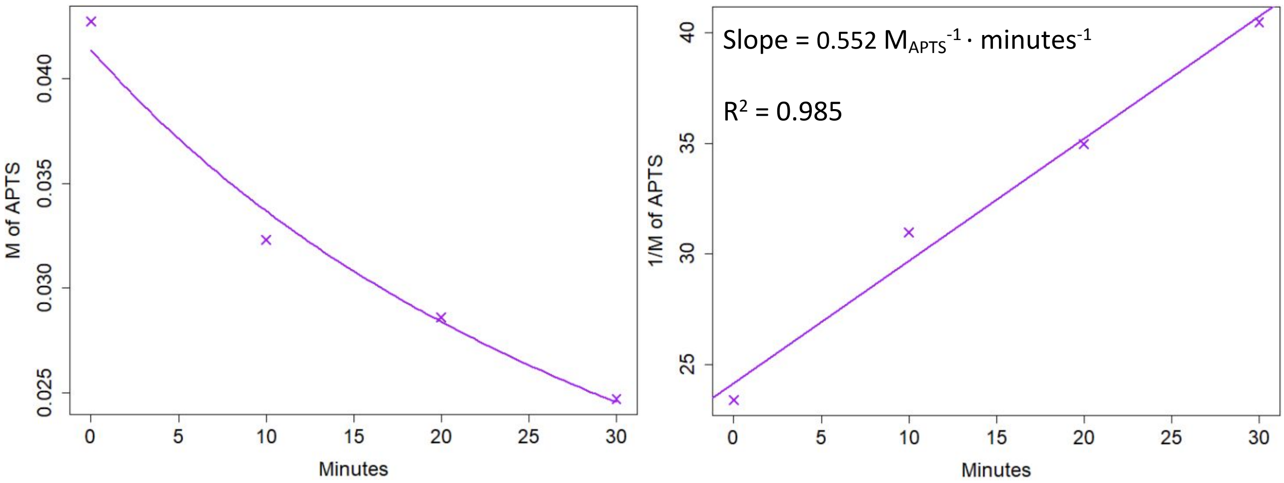

Figure S11. M of APTS consumed over time by grafting reaction in β-pinene.

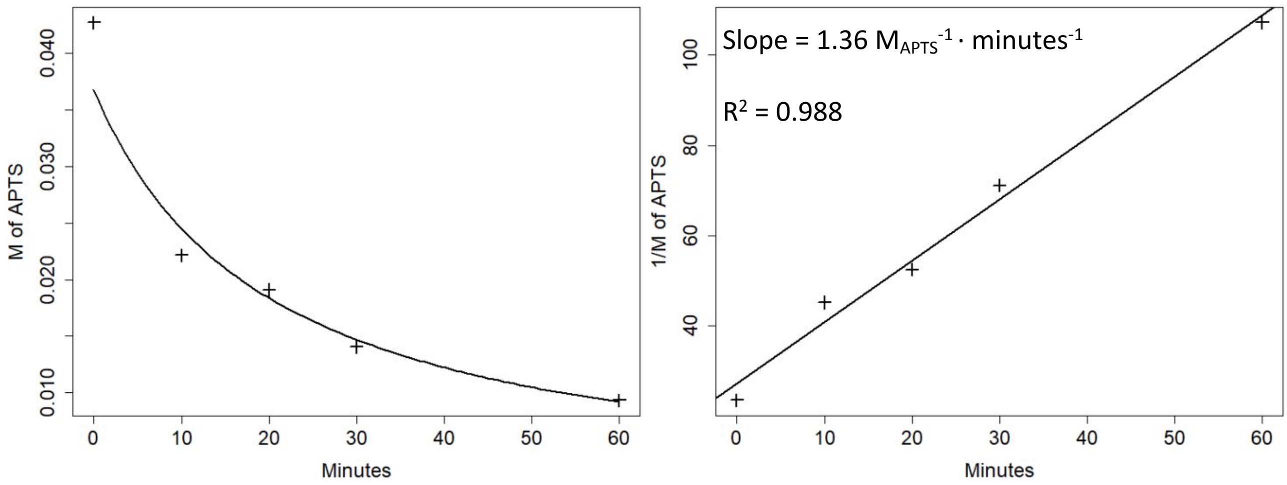

Figure S12. M of APTS consumed over time by grafting reaction in α-pinene.

FT-IR spectroscopy

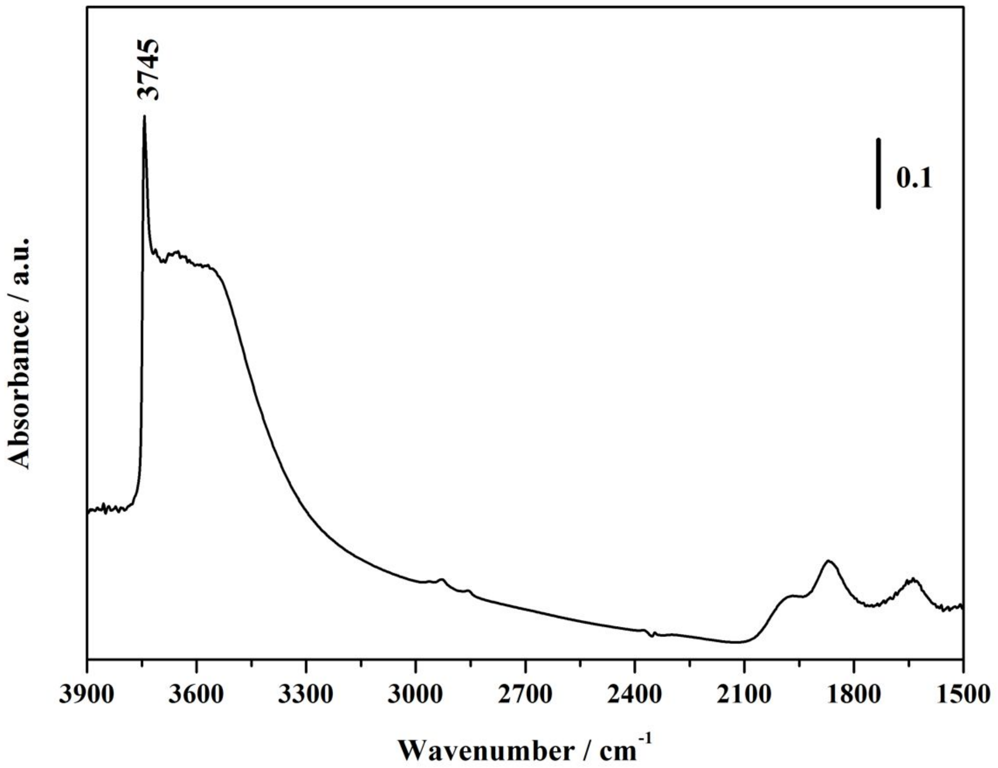

Figure S13. FTIR spectrum of MCM-41 upon treatment for 1 hour at 180°C to remove physisorbed water.
